# Dermotaxis v/s loop suture technique for closure of fasciotomy wounds: a study of 50 cases

**DOI:** 10.1007/s11751-017-0299-1

**Published:** 2017-10-16

**Authors:** Naveen Mittal, Robin Bohat, Jagandeep Singh Virk, Payal Mittal

**Affiliations:** 1 #3732 Sector 46-c, Chandigarh, India; 2 Government Medical College Hospital, Sector-32, Chandigarh, India

**Keywords:** Fasciotomy, Wound closure, Inexpensive, Dermotaxis, Loop suture technique

## Abstract

Fasciotomy incisions lead to large, unsightly, chronic wounds after surgical intervention. Classic management was to use split-thickness skin grafts, but this leads to insensate skin with reports that as many as 23% of patients are dissatisfied by the appearance of the wound. Since no skin loss has occurred with the fasciotomy incision, utilizing the dermal properties of creep, stress relaxation and load cycling, closure can be achieved in a better way. We describe using dermotaxis for skin edge approximation that is done using inexpensive equipment available readily in any standard operating room. Twenty-five patients had fasciotomy wounds closed either by dermotaxis or a loop suture technique with the inclusion criteria being closed fractures, no concomitant skin loss, fracture-related compartment syndrome and fasciotomy performed within 36 h. The fasciotomy incision was closed in a single stage by loop suture technique or gradually by dermotaxis once the oedema had settled between 3 and 5 days. Results were graded as excellent if approximation could be achieved, good if sutures had to be applied for protective care and poor if wounds needed to be skin-grafted. In the dermotaxis group, results were excellent in 15, good in 8 and poor in 2 cases. In the loop suture technique group, results were excellent in 20, good in 4 and poor in 1 case. Dermal apposition using inexpensive, readily available equipment is an alternative method for closure of fasciotomy wounds. If limb oedema has settled sufficiently, closure using a loop suture can be done in a single stage. If the limb remains oedematous, gradual closure can be done using dermotaxis.

## Introduction

Compartment syndrome was defined by Mubarak [1] as an elevation of the interstitial pressure in a closed osseofascial compartment that results in microvascular compromise. Early recognition and treatment via surgical decompression are paramount. However, the fasciotomy incisions can lead to large, unsightly, chronic wounds after surgical intervention [2]. The classic management of fasciotomy wounds was split-thickness skin graft [3, 4]. In a study involving 60 patients who underwent fasciotomy wound closure with split skin grafting, Fitzgerald et al. [3] reported that 23% were upset by the appearance of the wound, 28% changed hobbies and 12% changed occupation. Since no skin loss has occurred with the fasciotomy, several authors [4–6] have considered dermatotraction to gradually or acutely close fasciotomy wounds [6]. If skin is stretched with a constant force, it will expand with time as long as it is kept under tension, a phenomenon known as mechanical creep [7]. If skin is stretched to a constant distance, it will expand and lead to a decrease in the force or tension on the skin with time, a phenomenon known as stress relaxation [7]. Recently, load cycling has been identified as another skin stretching technique based on creep. Load cycling is the incremental elongation of skin when intermittent tension is applied. Tension applied for 3 min is followed by a load free period of 30–60 s. The same cycle is continued until the desired elongation is achieved [8, 9]. Our hypothesis was that closure of fasciotomy wounds by dermatotraction could be performed in a staged fashion or in a single stage, using inexpensive equipment available readily in any standard operating room.

## Materials and methods

**Fig. 1:**
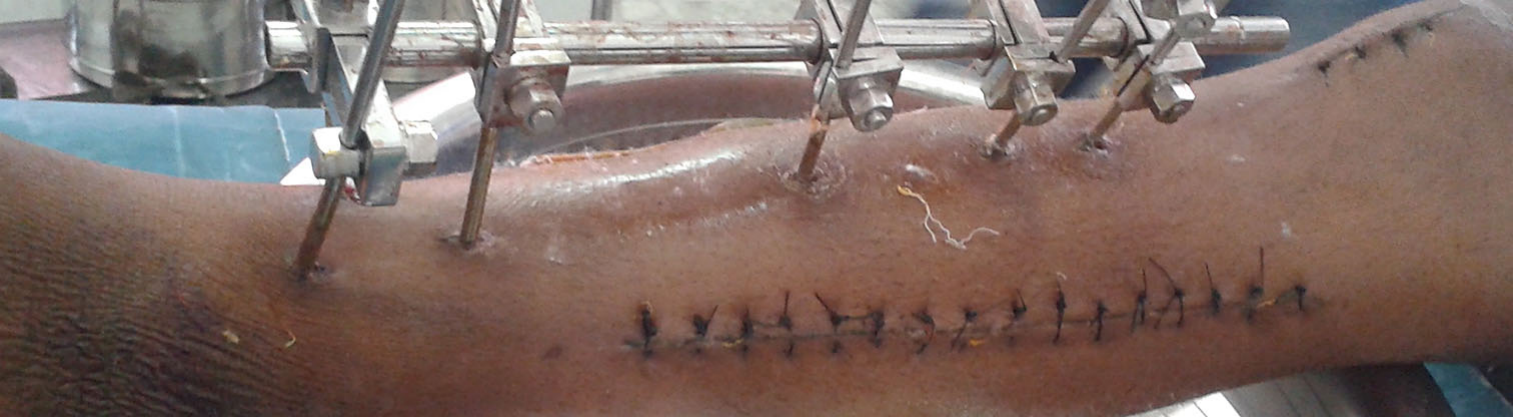
Showing closure of posteromedial incision by Vicryl no. 1 suture to the subcutaneous tissue and mattress sutures to the skin with Ethilon 2-0

There were 50 patients in total. There were two groups of twenty-five patients each, all with fasciotomy wounds, that had closure either by dermotaxis or the loop suture technique. The inclusion criteria were fasciotomies done for compartment syndrome after closed fractures, without concomitant skin loss, done within 36 h; the exclusion criteria were open fractures, skin loss, non-fracture-related compartment syndrome and presentation after 36 h. The diagnosis of compartment syndrome was made on clinical examination. The type of assisted closure, debridement repeated before final closure, need for skin graft and evidence of infection were recorded. We used the standard two-incision technique for fasciotomy of the leg using the posteromedial and anterolateral incisions. The closure of posteromedial incision was carried out using Vicryl (No. 1) sutures to the subcutaneous tissue and mattress sutures for the skin with Ethilon (No. 2-0) (Fig. 1). The wound on the lateral side was closed either by dermotaxis or a loop suture technique.

**Fig. 2:**
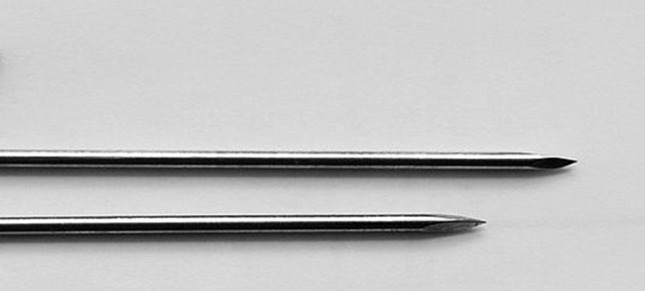
Showing dermotaxis apparatus consisting of K-wires

**Fig. 3:**
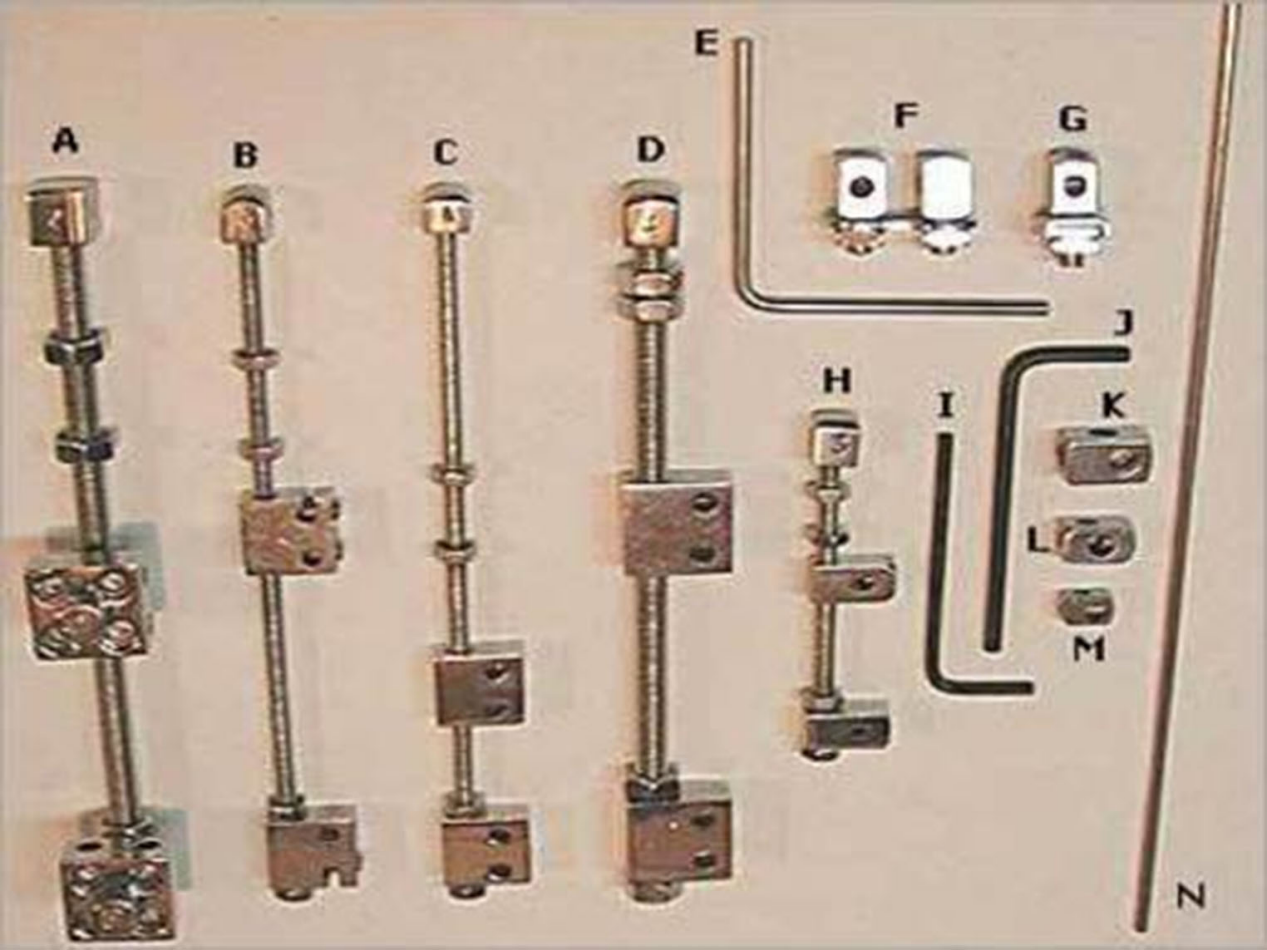
Showing dermotaxis apparatus consisting of compression knob

**Fig. 4:**
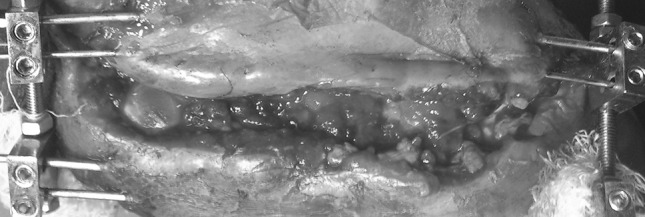
Showing wound after application of dermotaxis

In dermotaxis (Singh’s skin traction) method, two parallel Kirschner wires (1.5 mm) are passed through the dermis on either side of the wound margins and interconnected by compression device consisting of threaded rod having two blocks and compression knob. Gradual compression was applied daily at the rate of 1 turn/12 h on both sides of the wound (Figs. 2, 3, 4).

**Fig. 5:**
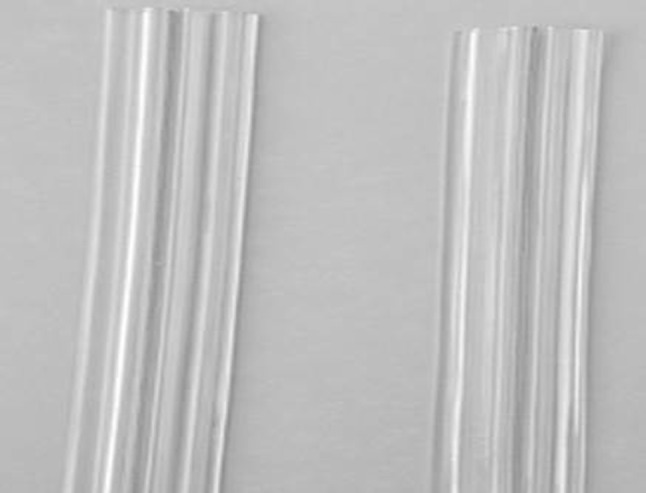
Showing corrugated drains

**Fig. 6:**
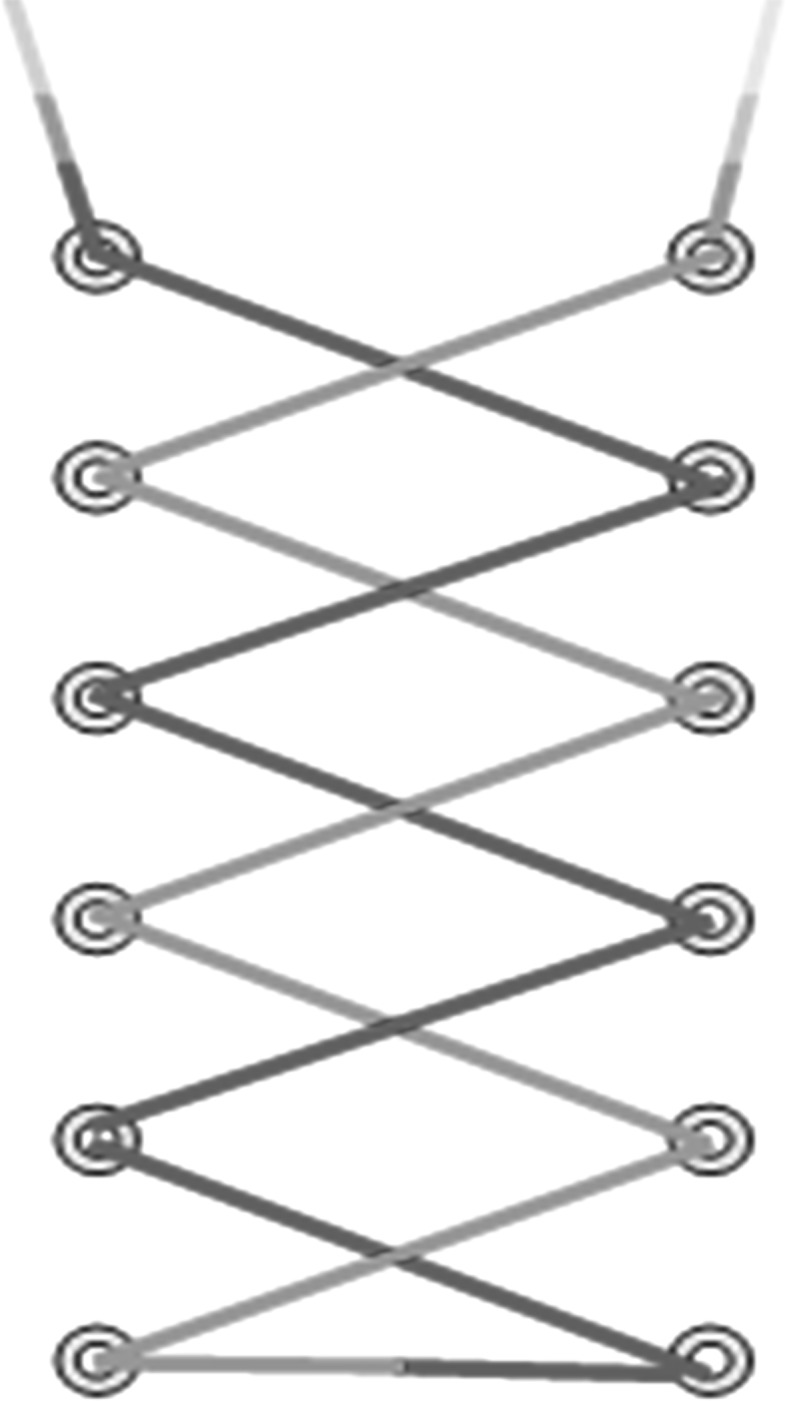
Showing loop suture technique

**Fig. 7:**
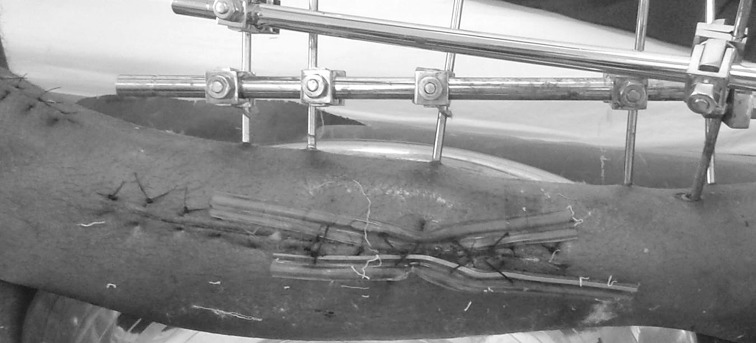
Showing complete approximation of the wound achieved with loop suture technique

The loop suture technique involves using corrugated drains and Ethilon (No. 1) suture. It is an extension of the purse string suture technique where a surgical suture is passed as a running stitch in and out along the edge of a wound in such a way that when the ends of the suture are drawn tight the wound is closed. Two corrugated drains (1, 2) are anchored to the skin adjacent to the fasciotomy incision using Ethilon (No. 1) suture. The sutures are then passed from one edge of the wound through the skin and corrugated drain to the other in an alternating fashion (Figs. 5, 6, 7). A moist dressing was applied, and the wound was left for 2 days to allow the skin to accommodate the approximation obtained.

## Results

**Fig. 8:**
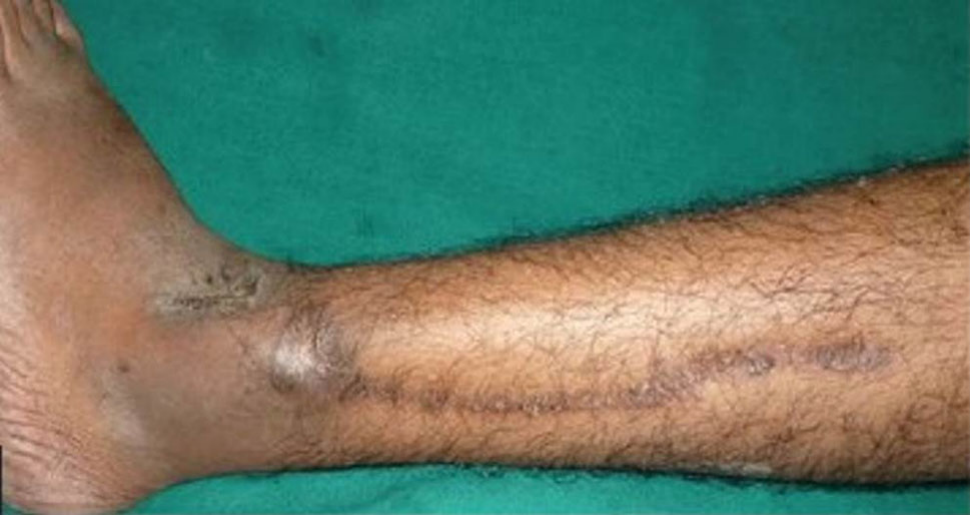
Showing complete closure of wound after dermotaxis

**Fig. 9:**
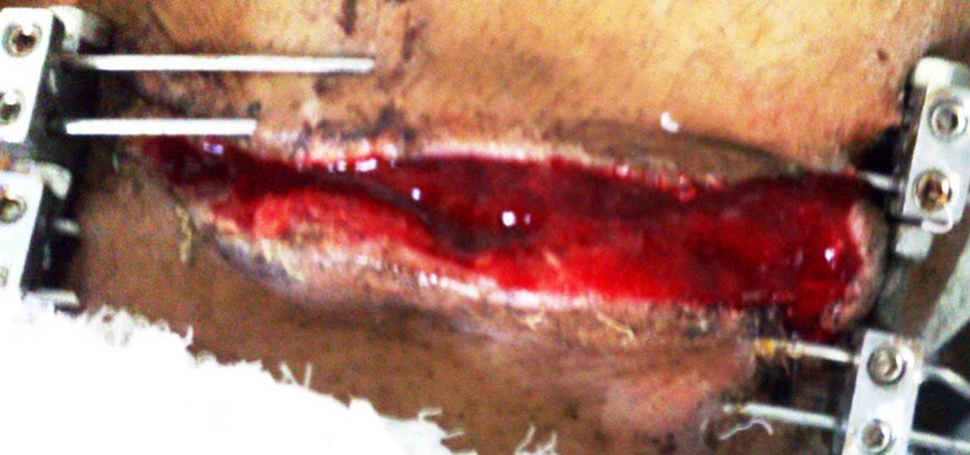
Showing maximum approximation achieved with dermotaxis, after which suture applied for approximation

**Fig. 10:**
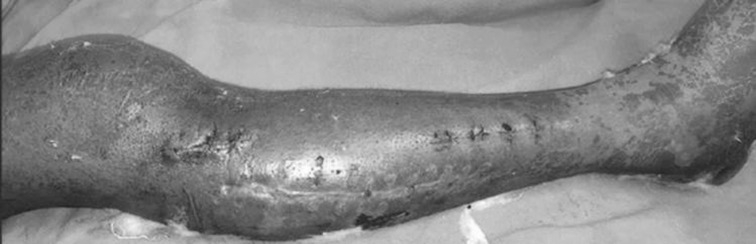
Showing complete closure after loop suture technique

The ages ranged from 21 to 60 years. The results were graded as excellent if approximation could be achieved, good if a secondary procedure, e.g. sutures had to be applied for protective care, and poor if the wounds had to be skin-grafted. In the dermotaxis group, results were excellent in 15, good in 8 and poor in 2 cases. In the loop suture technique, group results were excellent in 20, good in 4 and poor in 1 case (Figs. 8, 9, 10).

The results of the two techniques are compared in Table 1.

**Table 1:** Comparison of results of dermotaxis and loop suture technique

Technique	Dermotaxis	Loop suture
Number of patients	25(male 16; female 9)	25 (male 20; female 5)
Average dimensions of wound	18 × 8 cm	15 × 6 cm
Average closure time	12 Days	10 Days
Management	Daily tightening	Wound care
Limitations	K-wire cutout	Excessive skin tension
Cost	Rs 300/-($5)	Rs 100/-($2)
Number of tightening sessions	5–7	2–3 (If required)
Debridement	2 patients	4 Patients
Complications	Infection—3 patients	Infection—1

## Discussion

The various techniques of fasciotomy wound closure are:

- Split-thickness skin grafting.
- Healing by secondary intention.
- Vacuum-assisted closure (VAC).
- Dermal apposition.

These techniques each have advantages or disadvantages as compared with other techniques, including time to wound closure, cost and complications.

### Split skin grafting

Skin grafting is the most common procedure to close fasciotomy wounds [10]. The repair is tension free but requires another operative procedure, creating another wound [11, 12]. The overall cover is thin, producing an unappealing scar aesthetically that is without sensation [11, 12].

### Secondary intention

Secondary intention uses skin contraction to heal the fasciotomy wound [11]. It is free from the complications associated with grafting, but complete healing requires an average time of 3–4 months with dressings to be changed 1–3 times daily [11]. The normalization of the scar contour may take 4 years which is impractical today [11].

### Vacuum-assisted closure (VAC)

Vacuum-assisted closure(VAC) uses negative pressure to exploit the elastic properties of skin [10]. The negative pressure created reduces oedema, promotes blood flow, lowers bacterial count and increases the tissue granulation rate to create a wound that is more conducive to closure [10]. However, the daily cost of VAC is approximately $174 with the dressing changes every 2–3 days [13–15]. In addition, VAC may fail to provide adequate skin edge approximation for final closure with 5–10% of cases requiring a secondary procedure [16]. Patients who underwent NPWT-assisted closure were at higher risk for skin grafting than patients who underwent vessel loop closure. The overall time to closure was greater than loop suture technique [13, 17].

### Dermal apposition

The technique of dermatotraction for closure of fasciotomy wounds was first described by Cohn in 1986 [18]. In the literature, innovative techniques focusing on dermal apposition can be subdivided into closure in a single stage using inexpensive equipment and closure by gradual compression. The technique of closure by gradual compression is further subdivided into the expensive and inexpensive techniques.

The comparison of the expensive techniques is highlighted in Table 2. These have the advantage of a tension control mechanism, but cost and availability are against use as a standard technique.

**Table 2:** Dermal apposition using expensive techniques

Author	Device	Advantages	Disadvantages
Hirshowitz et al. [5]	Sure closure device	1. Can measure the tension across the wound edges	1. Not readily available
			2. Expensive ($300–$500)
McKenney et al. [19]	STAR (suture tension adjustment reel)		1. Expensive
Janzing and Broos [6]	Marburger skin approximation system		1. Not readily available
			2. Expensive
Barnea et al. [4]	Wisebands device	1. Tension feedback control mechanism to safeguard against excessive skin tensioning	1. Not readily available
			2. Expensive
Taylor et al. [20]	Skin anchors	2. Evenly distributed force over the full length of the wound	1. Not readily available
			2. Expensive
Medina et al. [12]	Silver bullet wound closure device (SBWCD)		1. Daily tightening
			2. Scar tenderness
			3. Numbness of extremity
			4. Expensive($575)
Geertruida et al. [16]	Ty-raps		1. Not readily available

The comparison of the inexpensive techniques is highlighted in Table 3. The inexpensive techniques, the shoelace technique and the peadiatric urinary catheter technique, both have the disadvantage of point loading as there is stretching on the skin at a point and are labour-intensive requiring the patient to be admitted to hospital. In contrast, our technique of dermotaxis creates uniform tension all across the wound and is not as labour-intensive as the gradual tightening can be performed at home by the patient as with adjustments to an Ilizarov fixator.

**Table 3:** Dermal apposition using inexpensive techniques

Author	Device	Advantages	Disadvantages
Zorrila et al. [21]	Shoelace	1. Readily available	3. Tightening every 48 h
		2. Inexpensive	4. Scar contracture
Ravinder et al. [22]	Dermotaxis (Singh’s skin traction)	1. Wounds with exposed bone	1. K-wire cutout
Eid et al. [23]	Paediatric urinary catheters + skin staples	1. Readily available	1. Point loading on the staples may lead to their failure
		2. Inexpensive	2. No safe mechanism against excess tension
Kakagia et a [13]	Shoelace	1. Readily available	3. Daily tightening
		2. Inexpensive	4. Replacement of device
Our study	Dermotaxis	1. No point loading	1. Sutures to approximate wound
		2. Postop easy	
		3. Readily available	
			2. K-wire cutout
		4. Inexpensive	

### Single closure

Chiverton and Redden used a subcuticular Prolene (No. 2-0) suture in 6 patients achieving good results in 4 patients. One intra-operative complication was encountered where an attempt to close the lateral wound led to breaking of the suture which had then to be replaced [24]. Marek et al. used spinal needle and towel clips for approximation in a case report. He used capillary refill for assessing skin vascularity, but this does not provide an assessment of the architecture of dermis; the study was limited to a single case [7]. Our loop suture technique uses the same concept as load cycling. The good and poor results were attributed to the thinning properties of skin at the rate of 6% per decade in addition to the dimensions of the original wound [7]. Caution is needed with these techniques as there is a potential risk of re-elevating compartment pressure; additionally, the excessive amount of traction may lead to blanching or impairment of skin vascularity.

The results of dermatotraction have been shown to be superior as it provides a better cosmetic appearance, sensate skin, avoids donor site morbidity and is easy to apply even in centres with limited resources. However, the disadvantages are that application has to await the settling of limb oedema after compartment syndrome, which may require three to five days postoperatively. When these devices are applied with oedema present, the tension created by the combination of the two can cause skin necrosis and a recurrence of the compartment syndrome. Therefore, close monitoring of the limb during closure is required.

## Conclusion

Dermal apposition using inexpensive, readily available equipment is a good method for closure of fasciotomy wounds. Both the methods have significant advantages to other techniques. If the reduction of limb oedema permits closure using the loop suture technique, then closure can be done in a single stage. If this oedema is still present, then gradual closure is done using dermotaxis. Despite its disadvantages, split skin grafting cannot be excluded totally given the ease of the procedure and its established role for wound closure.
